# Diagnostic Accuracy of Dual-Energy Computed Tomography for Bowel Necrosis in Acute Abdomen With Bowel Ischemia

**DOI:** 10.7759/cureus.81057

**Published:** 2025-03-23

**Authors:** Yosui Higuchi, Tatsuya Watanabe, Atsushi Tabeta, Hidetoshi Yamana, Yoshihiro Tanaka, Yusuke Tsutsumi

**Affiliations:** 1 Department of Emergency Medicine, National Hospital Organization Mito Medical Center, Ibaraki, JPN; 2 Department of Emergency and Critical Care Medicine, University of Tsukuba Hospital, Tsukuba, JPN; 3 Department of Surgery, National Hospital Organization Mito Medical Center, Ibaraki, JPN; 4 Department of Radiology, National Hospital Organization Mito Medical Center, Ibaraki, JPN

**Keywords:** acute abdomen, acute care surgery, computed tomography, diagnostic test accuracy, emergency medicine

## Abstract

Introduction

Immediate diagnosis of bowel necrosis in acute abdominal conditions is essential for proper treatment. Dual-energy computed tomography (DECT) has recently emerged as a tool to assess intestinal viability in bowel ischemia. However, DECT's diagnostic accuracy for bowel necrosis in clinical practice remains undefined.

Methods

This single-center retrospective cohort study at a Japanese tertiary care hospital assessed DECT's diagnostic accuracy for bowel necrosis. We included patients who underwent emergency operations for abdominal conditions with bowel ischemia from April 2013 to March 2022. Patients without DECT were excluded. The reference standard was bowel necrosis determined by surgical findings. Four evaluators (two residents and two specialists) independently reviewed DECT images blinded to confirmed diagnoses. We calculated pooled and individual sensitivity, specificity, likelihood ratios, and accuracy.

Results

Twenty-eight patients were included. Pooled sensitivity and specificity were 0.65 (95% confidence interval (CI), 0.52-0.77) and 0.83 (95% CI, 0.70-0.92), respectively. Residents demonstrated higher sensitivity than specialists. Pooled positive and negative likelihood ratios were 3.76 (95% CI, 2.02-7.00) and 0.42 (95% CI, 0.29-0.61). Overall accuracy was 0.73 (95%CI, 0.64-0.81). Inter-evaluator agreement was moderate (Fleiss' kappa, 0.42).

Conclusion

DECT demonstrated moderate diagnostic accuracy for bowel necrosis in this Japanese tertiary care setting. While clinically valuable, DECT's diagnostic capability was not definitive. To optimize DECT's potential, future research should employ disease-specific image reconstruction techniques and provide evaluators with specialized DECT interpretation training.

## Introduction

Bowel ischemia can be caused by several acute abdominal conditions, including acute mesenteric ischemia, strangulated small bowel obstruction, and incarcerated hernia [1-3]. For bowel ischemia, immediate diagnosis of bowel necrosis is essential for proper treatment. Currently, single-energy computed tomography (CT) with contrast medium is the standard diagnostic tool. In patients suspected of having bowel ischemia, a CT scan is typically performed before surgery. The CT scan allows clinicians to make an early diagnosis of bowel ischemia. If ischemia is diagnosed, the majority of cases are surgical indications, but unlike reversible ischemia, irreversible bowel necrosis requires bowel resection. CT is used to determine whether ischemia is reversible or irreversible before surgery and to identify the location and extent of bowel necrosis [4]. Bowel necrosis is associated with higher mortality [5]. Therefore, CT serves as a tool to provide important information to surgeons for selecting appropriate strategies and assessing the perioperative risk of patients [6]. However, previous studies have shown that its diagnostic accuracy, especially sensitivity, is suboptimal, reporting around 0.3-0.5 [7-9].

Recently, dual-energy CT (DECT) has been introduced to assess intestinal viability in cases of bowel ischemia. DECT utilizes two different X-ray energy levels to obtain images [10,11]. Conventional single-energy CT operates at one energy level and cannot differentiate between materials with the same attenuation coefficient. Greater attenuation differences of DECT at low energy levels help distinguish between infarcted and perfused bowel segments. Therefore, DECT may provide a more accurate diagnosis of intestinal necrosis than single-energy CT, potentially replacing SECT as the preferred diagnostic method [12].

However, the evidence of the diagnostic accuracy of DECT for bowel necrosis in actual clinical practice is still insufficient [13]. In this study, we report the diagnostic accuracy of DECT for bowel necrosis in the setting of a Japanese single tertiary care center. We aim to evaluate the diagnostic accuracy of DECT for identifying bowel necrosis in patients with bowel ischemia, which contributes to the accumulation of evidence regarding its potential to replace SECT in clinical practice.

## Materials and methods

Study design and setting

This study was a single-center retrospective cohort study conducted at the NHO Mito Medical Center, one of the tertiary care centers in Ibaraki, Japan. We followed the Standards for the Reporting of Diagnostic Accuracy Studies (STARD) 2015 guidelines (see Appendix A) [14]. This study was approved by the institutional review board of the NHO Mito Medical Center (reference numbers: 2022-17 and 2024-13).

Study population

We consecutively included patients who underwent emergency operations from April 2013 to March 2022 for one of the abdominal emergency conditions with bowel ischemia, which may cause bowel necrosis as follows: strangulated bowel obstruction, incarcerated hernia, and acute mesenteric ischemia. We excluded patients who did not receive DECT.

Index test

The index test was DECT. We utilized a second-generation dual-source 128-slice multi-detector computed tomography (Somatom Definition Flash, Siemens Healthcare). Our detailed CT protocol for acute abdomen with bowel ischemia is provided in Appendices B-D.

Four evaluators retrospectively reviewed the DECT images and diagnosed whether there was intestinal necrosis. Evaluators consisted of one emergency medicine resident in the third year of training (Evaluator 1), one surgical resident in the first year of surgical training following two years of emergency medicine residency (Evaluator 2), one board-certified specialist in both emergency medicine and radiology with 13 years of clinical experience (Evaluator 3), and one board-certified specialist in both emergency medicine and surgery with 20 years of clinical experience (Evaluator 4). The evaluators were selected from members of the Departments of Emergency Medicine and Surgery, taking into account their clinical experience and specialist qualifications. The evaluators were asked to assess the images independently without any clinical information such as the patients' characteristics and confirmed diagnoses including the presence or absence of bowel necrosis. This was because we specifically intended to examine the diagnostic accuracy of DECT based on image interpretation alone. They were asked to make assessments based on their own clinical experience. Actually, they made comprehensive assessments considering various signs such as poor contrast enhancement of the bowel wall or intramural gas; however, we did not pre-defined the diagnostic criteria to closely resemble real-world clinical practices, where different evaluators may emphasize different signs. Quality of assessment was ensured by selecting evaluators who are physicians specializing in emergency medicine, surgery, or radiology. The evaluation utilized three imaging phases: non-enhanced CT, contrast-enhanced arterial phase CT, and contrast-enhanced venous phase CT. This study aimed to assess the diagnostic accuracy of DECT in actual clinical practice when using the standard three-phase protocol typically employed for single-energy CT without additional specialized image reconstruction.

Reference standard

The reference standard was bowel necrosis requiring resection, as determined by surgical findings. While pathological findings provide more reliable judgment, patients who did not undergo intestinal resection obviously lack pathological specimens. Therefore, we defined the bowel necrosis requiring resection as our reference standard, representing the best available alternative. The indication of resection is typically made by two or three surgeons participating in the operation. Because this study was retrospective, the surgeons reviewed the DECT before surgery as usual in clinical practice.

Statistical analyses

We presented the descriptive data on the baseline characteristics of the included patients as the median and interquartile range for continuous variables and the number and percentage for categorical variables.

For diagnostic accuracy, we calculated the sensitivity, specificity, positive and negative likelihood ratio, and accuracy by combining the judgments of all the evaluators as well as of each evaluator. We examined the reproducibility between evaluators using Fleiss' kappa coefficient [15]. We categorized agreement as follows: kappa < 0 as poor, 0.01-0.20 as slight, 0.21-0.40 as fair, 0.41-0.60 as moderate, 0.61-0.80 as substantial, and 0.81-1.00 as almost perfect agreement [15]. We performed all analyses using Stata software (version 14.0, Stata Corporation, College Station, Texas, USA) and epiR (version 2.0.80) package of R (version 4.4.2) [16]. All statistical analyses were conducted with a two-sided alpha error of 5%.

## Results

Baseline characteristics

**Figure 1:**
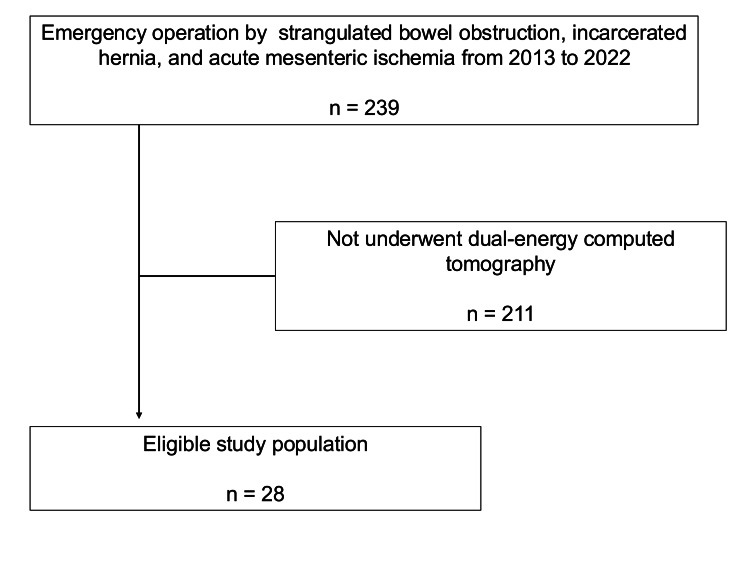
Patient flow.

A total of 239 patients underwent emergency abdominal operations by strangulated bowel obstruction, incarcerated hernia, and acute mesenteric ischemia during the study periods. Out of the total, 211 participants were excluded for not undergoing DECT. As a result, 28 patients were included in the eligible study population (Figure 1). Table 1 reveals the baseline characteristics of the included patients. The mean age was 76 (interquartile range, 69-83), and 15 (54%) patients were male. The most common cause of the disease was strangulated intestinal obstruction. Bowel necrosis was confirmed in 15 patients (54%).

**Table 1:** Baseline characteristics of the included patients. IQR, Interquartile range

Characteristic	n = 28
Age, median (IQR)	76 (69-83)
Gender, n (%)	
Male	15 (54%)
Female	13 (46%)
Vital signs on admission	
Systolic blood pressure, median (IQR)	138 (127-156)
Heart rate, median (IQR)	80 (75-94)
Glasgow Coma Scale, n (%)	
13	1 (3·6%)
14	2 (7·1%)
15	25 (89%)
Body temperature, median (IQR)	36.7 (36.2-37.0)
Postoperative diagnosis, n (%)	
Strangulated bowel obstruction	20 (71%)
Incarcerated hernia	5 (18%)
Acute mesenteric ischemia	3 (11%)
Necrosis, n (%)	15 (54%)

Diagnostic accuracy of DECT for bowel necrosis

The grand total sensitivity and specificity were 0.65 (95% confidence interval (CI), 0.52-0.77) and 0.83 (95%CI, 0.70-0.92), respectively (Table 2, Appendix E). Among the evaluators, the sensitivity ranged from 0.47 to 0.80, while the specificity ranged from 0.77 to 0.92. Resident physicians showed higher sensitivity than specialist physicians in the setting of this study. Fleiss’ kappa coefficient between evaluators was 0.42, indicating moderate judgment agreement. The pooled positive and negative likelihood ratios were 3.76 (95%CI, 2,02-7.00) and 0.42 (95%CI, 0.29-0.61). Overall, the accuracy was 0.73 (95%CI, 0.64-0.81).

**Table 2:** Diagnostic accuracy of DECT DECT, dual-energy computed tomography; EM, emergency medicine

	Sensitivity (95%CI)	Specificity (95%CI)	Positive likelihood ratio (95%CI)	Negative likelihood ratio (95%CI)	Accuracy (95%CI)
Grand total evaluators	0.65 (0.52-0.77)	0.83 (0.70-0.92)	3.76 (2.02-7.00)	0.42 (0.29-0.61)	0.73 (0.64-0.81)
Evaluator 1 (EM resident)	0.80 (0.52-0.96)	0.92 (0.64-1.00)	10.40 (1.56-69.53)	0.22 (0.08-0.60)	0.86 (0.67-0.96)
Evaluator 2 (Surgery resident)	0.73 (0.45-0.92)	0.77 (0.46-0.95)	3.18 (1.13-8.98)	0.35 (0.14-0.84)	0.75 (0.55-0.89)
Evaluator 3 (EM / radiology specialist)	0.47 (0.21-0.73)	0.77 (0.46-0.95)	2.02 (0.65-6.26)	0.69 (0.40-1.21)	0.61 (0.41-0.78)
Evaluator 4 (EM / surgery specialist)	0.60 (0.32-0.84)	0.85 (0.48-0.98)	3.90 (1.02-14.90)	0.47 (0.24-0.92)	0.71 (0.51-0.87)

## Discussion

In this study, we assessed the diagnostic accuracy of DECT as a potential replacement for current SECT protocols in routine clinical practice. As a result, we found a sensitivity of 0.65 and specificity of 0.83 of DECT for bowel necrosis among acute abdominal conditions with bowel ischemia. Both positive and negative likelihood ratios showed small changes in the pretest to posttest necrosis probability [17]. Reproducibility between evaluators was moderate. According to the results, simply replacing SECT with DECT does not provide a level of diagnostic accuracy that is clinically definitive.

CT imaging plays a crucial role in preoperative diagnosis [6]. However, conventional CT demonstrates insufficient sensitivity for detecting intestinal necrosis [18]. DECT is expected to improve the diagnosis of intestinal necrosis compared to SECT [19, 20]. DECT can provide low-energy images than SECT, allowing substances containing iodine to be visualized with greater clarity [10]. This improved visualization has demonstrated higher diagnostic accuracy in previous animal studies [19]. Yet, evidence from real clinical practice remains insufficient. A previous study reported that DECT increased sensitivity without decreasing high specificity, evaluating acute mesenteric ischemia [21]. However, this study used matched controls in whom intestinal ischemia was not suspected, therefore, the results may be affected by spectrum bias. Our study examined the diagnostic accuracy for detecting bowel necrosis in patients with suspected bowel ischemia in line with real-world clinical settings. Therefore, we suppose that our study minimizes potential spectrum bias effects on our results. As a result, we found that the diagnostic accuracy for necrosis may be insufficient in this clinical context. This finding suggests that low-energy images alone may not provide clinically meaningful improvements in diagnostic accuracy. However, it is important to note that the low-energy setting used in this study was 100 keV. Accuracy might improve if lower energy settings of 40-50 keV were employed, as suggested by previous research [10, 22].

Furthermore, DECT offers numerous disease-specific reconstruction options for clinical applications [23]. For bowel ischemia, the iodine map and the iodine concentration in the bowel wall can be used to evaluate necrosis both subjectively and quantitatively [22, 24-26]. Another potential modality is virtual non-contrast imaging [27]. Previous studies have reported that enhancement can be detected more clearly when compared to non-enhanced images in conventional SECT [28]. Therefore, incorporating virtual non-contrast imaging may improve diagnostic accuracy. However, implementing these advanced methods requires both radiologists and evaluators to be familiar with the specialized methodology [23]. This technical expertise requirement presents a significant barrier to widespread adoption. In this study, we evaluated the diagnostic accuracy of DECT within the three-phase CT protocol used in routine clinical examinations. Therefore, we neither performed specialized DECT reconstructions nor provided evaluators with advanced training on specific DECT interpretation techniques. By providing specific reconstructed images for bowel ischemia or training specific DECT readings for evaluators, the diagnostic performance of DECT may be substantially increased.

In addition, we found that resident physicians showed higher sensitivity than specialist physicians in this setting. Residents, aware of their limited experience, may adopt a more cautious approach, classifying cases as positive even with minimal suspicion of necrosis to avoid missing critical findings. Conversely, specialists may rely more on their clinical experience and consider a broader differential diagnosis, potentially leading to a more conservative classification of equivocal cases.

Our study has several strengths. First, the topic is very relevant where the timely diagnosis of bowel necrosis and appropriate preoperative information for surgeons in acute abdominal conditions is essential to improve patient outcomes. Second, we examined the diagnostic accuracy using real-world data in a setting similar to a real clinical setting to provide insights into the practical application of DECT. Third, we involved diverse evaluators including both residents and specialists allowing for an assessment of DECT's performance across different experience levels.

On the other hand, our study has some limitations. First, as a single-center study conducted in Japan, its generalizability to other populations and healthcare settings may be limited which restricts the applicability of the results to broader populations. Second, the small sample size reduced statistical power and resulted in wide confidence intervals. A small sample size may induce larger random error but does not necessarily violate the validity of the study. Therefore, we believe the study still provides meaningful evidence despite this limitation. Further multi-center studies with larger sample sizes and optimized DECT protocols are needed to address these limitations. Third, inter-evaluator agreement was moderate indicating variability in interpretation, which could impact the reliability of the results. Fourth, the absence of specialized training for evaluators in DECT interpretation may have influenced diagnostic accuracy. In this study, we evaluated the diagnostic accuracy of DECT by analyzing images without utilizing any DECT-specific reconstruction methods or providing special training to evaluators. This approach was designed to simulate a realistic clinical scenario where DECT would directly replace SECT. Our findings indicate that simply substituting DECT for SECT does not significantly improve diagnostic accuracy. However, we acknowledge that certain potential advantages of DECT may not have been fully assessed within the constraints of our study design. Fifth, the exclusion of patients who did not undergo DECT may introduce selection bias, as the cohort may not represent all patients with bowel ischemia. Therefore, the generalizability of the results may be low.

## Conclusions

This study examined the diagnostic accuracy of DECT for bowel necrosis in patients with suspected intestinal ischemia. Our results demonstrated that DECT achieved moderate diagnostic performance when using standard three-phase protocols. While DECT proved clinically valuable, its diagnostic capability was not definitive in this context. To fully explore DECT's diagnostic potential, future studies should implement disease-specific image reconstruction techniques and evaluate performance after providing evaluators with specialized training in DECT image interpretation.

## Figures and Tables

**Figure 2:**
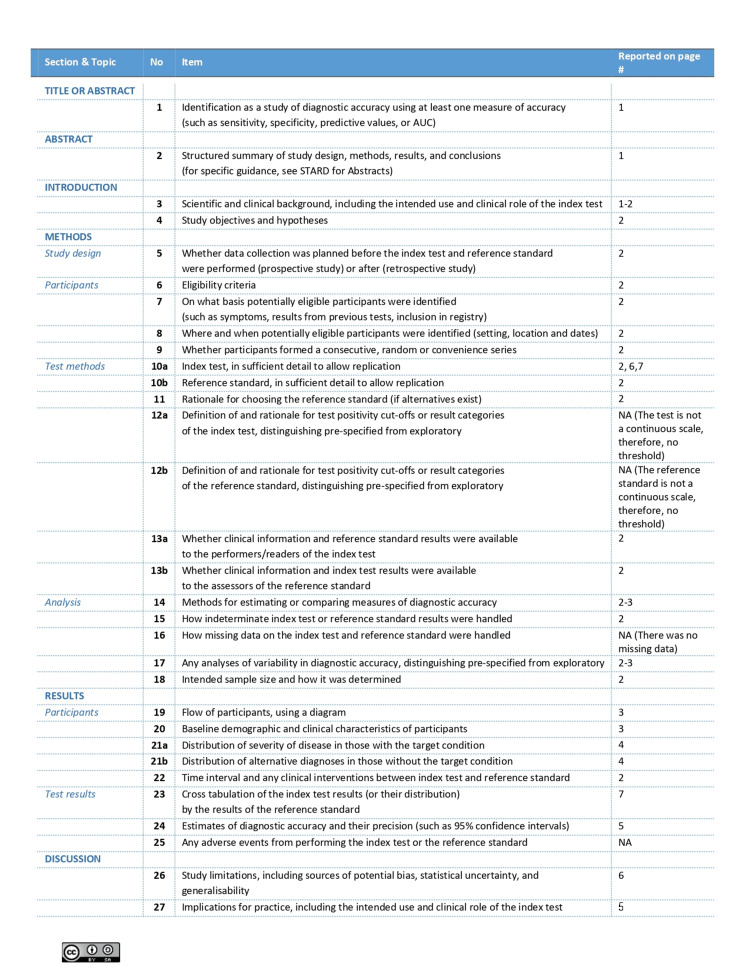
Standards for the Reporting of Diagnostic Accuracy Studies (STARD) 2015 guidelines (Part 1)

**Figure 3:**
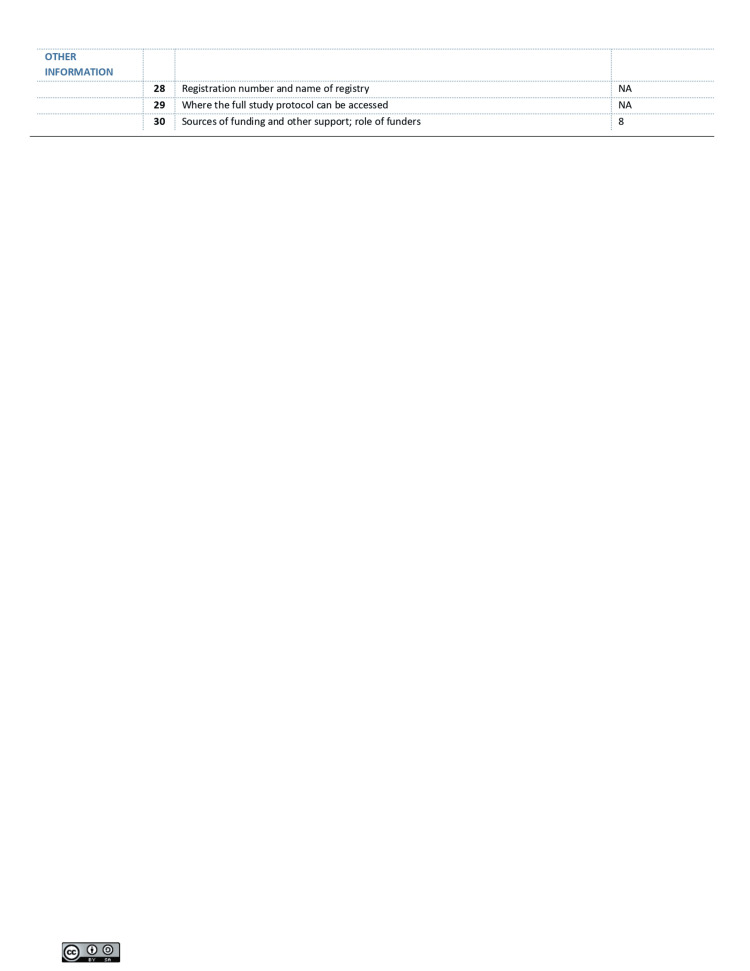
Standards for the Reporting of Diagnostic Accuracy Studies (STARD) 2015 guidelines (Part 2)

**Figure 4:**
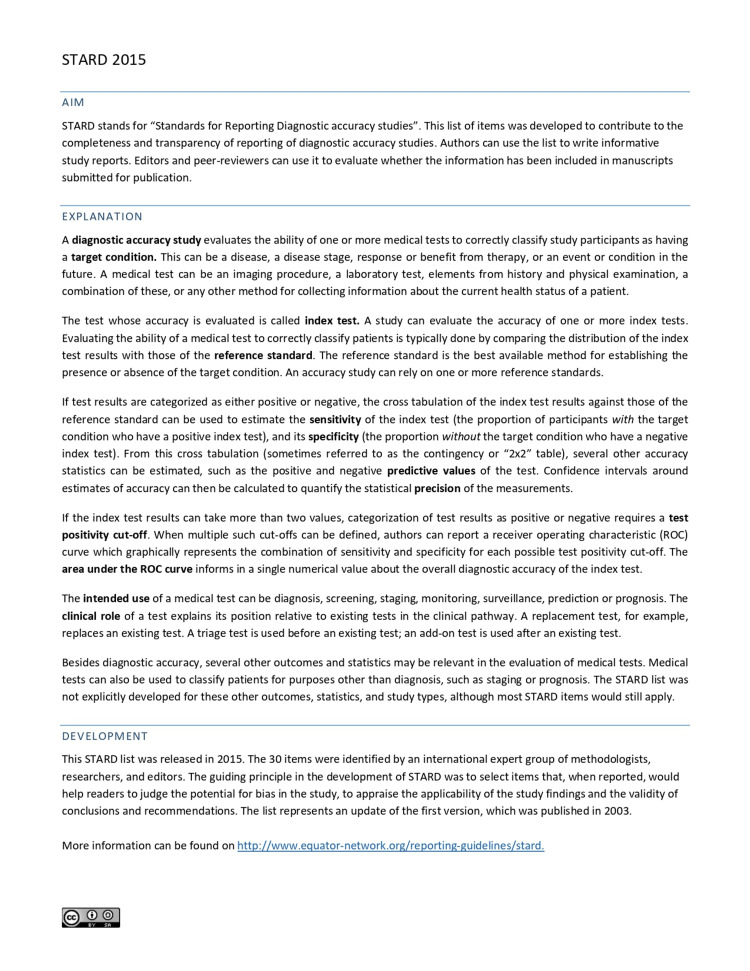
Standards for the Reporting of Diagnostic Accuracy Studies (STARD) 2015 guidelines (Part 3)

## Appendices

Appendix A

Appendix B

**Table 3:** CT protocol for the acute acdomen. SE, single energy; DE, dual energy

Parameter	Non-enhanced phase (SE)	Arterial phase (SE)	Venous phase (DE)
Detector configuration (mm)	128×0.625	128×0.625	32×0.625
Tube voltage (kV)	120	120	100/140
Reference mAs	120	135	115/89
Gantry rotation time (s)	0.5	0.5	0.5
Helical pitch	0.8	0.8	1.2
Field of view (cm)	50	50	50
Reconstructed section thickness (mm)	5.0	5.0	5.0
Reconstruction algorithm	Projection soft tissue	Projection soft tissue	Projection soft tissue
Kernel	B40f	B40f	B40f
Detector configuration (mm)	128×0.625	128×0.625	32×0.625

Appendix C

**Table 4:** Contrast injection protocol

Contrast injection protocol	
Contrast agent	Non-ionic iodinated contrast medium
Injection rate	3.0–5.0 mL/sec (depending on vascular access and patient condition)
Contrast dose	1.5 mL/kg (typically 80–150 mL)
Saline flush	20–50 mL (optional, improves contrast bolus)

Appendix D

**Table 5:** Imaging phases and timing ROI, region of interest

Imaging phases and timing	
Non-contrast phase	For detecting hemorrhage, calcifications, or certain pathologies.
Placement of ROI	Abdominal aorta (at the level of the celiac artery or upper abdominal aorta).
Trigger threshold	120 HU (depending on protocol and scanner settings)
Arterial phase	Scan triggered 16 seconds after reaching the threshold (used for vascular assessment, ischemia, or active bleeding)
Delayed phase	100 seconds after injection start (standard phase for most acute abdomen evaluations)

Appendix E

**Table 6:** Number of true-positive, false-positive, false-negative, and true-negative patients.

Evaluator	True positive	False positive	False negative	True negative
Grand total of evaluators	39	9	21	43
Evaluator 1	12	1	3	12
Evaluator 2	11	3	4	10
Evaluator 3	7	3	8	10
Evaluator 4	9	2	6	11
