# Correction: Diagnostic Accuracy of Dual-Energy Computed Tomography for Bowel Necrosis in Acute Abdomen With Bowel Ischemia

**DOI:** 10.7759/cureus.c219

**Published:** 2025-05-02

**Authors:** Yosui Higuchi, Tatsuya Watanabe, Atsushi Tabeta, Hidetoshi Yamana, Yoshihiro Tanaka, Yusuke Tsutsumi

**Affiliations:** 1 Department of Emergency Medicine, National Hospital Organization Mito Medical Center, Ibaraki, JPN; 2 Department of Emergency and Critical Care Medicine, University of Tsukuba Hospital, Tsukuba, JPN; 3 Department of Surgery, National Hospital Organization Mito Medical Center, Ibaraki, JPN; 4 Department of Radiology, National Hospital Organization Mito Medical Center, Ibaraki, JPN

This article has been corrected as follows. Yosui Higuchi’s affiliation has been updated to the following: Department of Emergency Medicine, National Hospital Organization Mito Medical Center, Ibaraki, JPN.

